# The CCCTC-binding factor CTCF represses hepatitis B virus enhancer I and regulates viral transcription

**DOI:** 10.1111/cmi.13274

**Published:** 2020-10-16

**Authors:** Valentina D’Arienzo, Jack Ferguson, Guillaume Giraud, Fleur Chapus, James M. Harris, Peter A. C. Wing, Adam Claydon, Sophia Begum, Xiaodong Zhuang, Peter Balfe, Barbara Testoni, Jane A. McKeating, Joanna L. Parish

**Affiliations:** 1 Nuffield Department of Medicine, University of Oxford, Oxford, UK; 2 institute of Cancer and Genomic sciences, College of Medical and Dental Sciences, University of Birmingham, Birmingham, UK; 3 CRCL INSERM and Cancer Research Center of Lyon (CRCL), Lyon, France

**Keywords:** CTCF, epigenetics, HBV, transcription insulation, transcription regulation

## Abstract

Hepatitis B virus (HBV) infection is of global importance with over 2 billion people exposed to the virus during their lifetime and at risk of progressive liver disease, cirrhosis and hepatocellular carcinoma. HBV is a member of the *Hepadnaviridae* family that replicates via episomal copies of a covalently closed circular DNA (cccDNA) genome. The chromatinization of this small viral genome, with overlapping open reading frames and regulatory elements, suggests an important role for epigenetic pathways to regulate viral transcription. The chromatin-organising transcriptional insulator protein, CCCTC-binding factor (CTCF), has been reported to regulate transcription in a diverse range of viruses. We identified two conserved CTCF binding sites in the HBV genome within enhancer I and chromatin immunoprecipitation (ChIP) analysis demonstrated an enrichment of CTCF binding to integrated or epi-somal copies of the viral genome. siRNA knock-down of CTCF results in a significant increase in pre-genomic RNA levels in de novo infected HepG2 cells and those supporting episomal HBV DNA replication. Furthermore, mutation of these sites in HBV DNA minicircles abrogated CTCF binding and increased pre-genomic RNA levels, providing evidence of a direct role for CTCF in repressing HBV transcription.

## 1 Introduction

Hepatitis B virus (HBV) infection is one of the world’s unconquered infections with an estimated 2 billion people exposed to the virus in their lifetime. HBV replicates in hepatocytes, and chronic infection can result in progressive liver disease, cirrhosis and hepatocellular carcinoma. HBV is a member of the *Hepadnaviridae* family and is classified into eight genotypes, A-H (McNaughton et al., 2019), that can associate with different clinical outcomes and treatment responses (Kao, Chen, Lai, & Chen, 2000). The HBV genome is a small, partially double-stranded relaxed circular DNA (rcDNA) of approximately 3.2 Kb. Following HBV entry into hepatocytes via the liver-specific bile-acid transporter, sodium taurocholate co-transporting polypeptide (NTCP) (Ni et al., 2014), rcDNA is released into the nucleus and is repaired by host DNA repair pathways to generate covalently closed circular DNA (cccDNA). This cccDNA persists in the nucleus as a long-lived nucleosome-associated minichromosome (Lythgoe, Lumley, McKeating, & Mathews, 2020) and is the transcriptional template for all viral RNAs (Hong, Kim, & Guo, 2017).

The HBV genome is transcribed by the host RNA polymerase II (RNA pol II) complex from four promoters (basal core promoter, BCP, Sp1, Sp2 and Xp) (Hong et al., 2017) that results in six major viral RNAs of increasing length with heterogeneous 5’ ends and a common polyadenylation signal (Stadelmayer et al., 2020). These RNAs include: pre-core (preC) that encodes e antigen (HBeAg); pre-genomic (pgRNA) that is translated to yield core protein (HBcAg) and polymerase; preS1, preS2 and S RNAs encoding the surface envelope glycoproteins and X transcript for the multi-functional x protein (HBx). Two viral enhancers regulate viral transcription: Enhancer I (EnhI) is located upstream of and partially overlaps the X promoter (Xp) and directs BCP activity (Hu & Siddiqui, 1991), which stimulates the production of both preC and pgRNAs. Enhancer II (EnhII) overlaps a large portion of BCP and stimulates activity of the distal Sp1 and Sp2 promoters, as well as the BCP and Xp (Yuh & Ting, 1990). The BCP encodes a negative regulatory element (NRE) that overlaps with Enh II (Sun et al., 2001) and has been reported to repress promoter activity. Encapsidated pgRNA is reverse-transcribed by the viral polymerase to generate new DNA genomes that can be re-imported to the nucleus to maintain the cccDNA pool or are enveloped and secreted as infectious particles (Urban, Schulze, Dandri, & Petersen, 2010), highlighting an essential role for the BCP in regulating viral replication.

HBV cccDNA is assembled into nucleosomes by cellular histones to form episomal chromatin (Levrero et al., 2009). The viral DNA is enriched with active epigenetic histone modifications including trimethylation of lysine 4 (H3K4Me3) and acetylation of lysine 27 on histone 3 (H3K27Ac) but devoid of repressive marks, such as trimethylation of lysine 27 on histone 3 (H3K27Me3) (Flecken et al., 2019; Tropberger et al., 2015). The overlap of active histone marks with RNA pol II occupancy suggests that viral transcription is regulated by epigenetic modification. In support of this, treating de novo infected primary human hepatocytes with inhibitors of the histone acetyltransferase p300/CBP reduces HBV RNA levels (Tropberger et al., 2015). Although the mechanisms underlying the epigenetic regulation of HBV cccDNA are not fully understood, several epigenetic modifiers are recruited to HBV cccDNA by HBx. As such, HBx behaves as a transcriptional regulator of both viral and cellular promoters (Guerrieri et al., 2017), and although HBx cannot bind to DNA directly, it can associate with components of the basal transcription machinery, transcription factors and transcriptional coactivators (Tang, Oishi, Kaneko, & Murakami, 2006). HBx coordinates the recruitment of the CBP/p300 and PCAF histone acetyl transferases (HAT) to cccDNA while facilitating the exclusion of histone deacetylases (HDACs) HDAC1 and Sirtuin 1 (Sirt1), resulting in hyperacetylation of cccDNA (Belloni et al., 2009; Chong et al., 2020). HBV transcription is dependent on an array of ubiquitous and liver-specific cellular transcription factors, including the liver-specific hepatocyte nuclear factors, 1 and 4 (HNF-1/4), and ubiquitously expressed octamer-binding protein 1 and specificity protein 1 (reviewed in Oropeza et al., 2020; Turton, Meier-Stephenson, Badmalia, Coffin, & Patel, 2020).

The genomes of metazoans are organised into megabase-sized regions termed topologically associated domains (TADs) that provide regulatory segmentation required for appropriate gene expression and replication. TADs are separated by regions enriched in binding sites of the ubiquitously expressed CCCTC-binding factor (CTCF), which stabilises chromatin loops by anchoring cohesin rings at the base of the loops (Rowley & Corces, 2018). Such spatial organisation can create epigenetic boundaries that separate transcriptionally active and inactive chromatin domains and control cis-regulatory elements, such as transcriptional enhancers. CTCF binds to tens of thousands of either ubiquitous or cell type specific consensus binding sites within the human genome, regulating both tissue-specific and developmental changes in gene expression (Chen, Tian, Shu, Bo, & Wang, 2012).

The occupancy of specific CTCF binding sites is dictated by chromatin accessibility and local epigenetic status (Kumar & Bucher, 2016). In addition to the organisation of chromatin domains, CTCF can function as a transcriptional repressor, or activator, by direct association with promoter proximal elements. CTCF was shown to act as a transcriptional repressor of the *c-myc* oncogene by creating a roadblock to RNA pol II (Filippova et al., 1996). Conversely, CTCF can physically associate with transcriptional regulators, such as the general transcription factor, TFII-I, to promote recruitment of the cyclin-dependent kinase 8, resulting in stimulation of RNA pol II activity (Pena-Hernandez et al., 2015). CTCF regulates the transcription (up or down) of evolutionarily distinct DNA viruses (Pentland & Parish, 2015) including: Kaposi sarcoma-associated herpesvirus; Epstein-Barr virus and herpes simplex virus (Chen et al., 2014; Kang, Wiedmer, Yuan, Robertson, & Lieberman, 2011; Lang et al., 2017; Washington, Musarrat, Ertel, Backes, & Neumann, 2018). We have demonstrated that CTCF recruitment to the human papillomavirus (HPV) genome negatively regulates early promoter usage via host cell differentiation-specific stabilisation of an epigenetically repressed chromatin loop (Paris et al., 2015; Pentland et al., 2018). However, a role in HBV transcription regulation has not yet been reported, herein we show that CTCF binds HBV DNA and acts as a repressor of viral transcription.

## 2 Results

### 2.1 CTCF binds HBV DNA at conserved sites within enhancer elements

**Figure 1:**
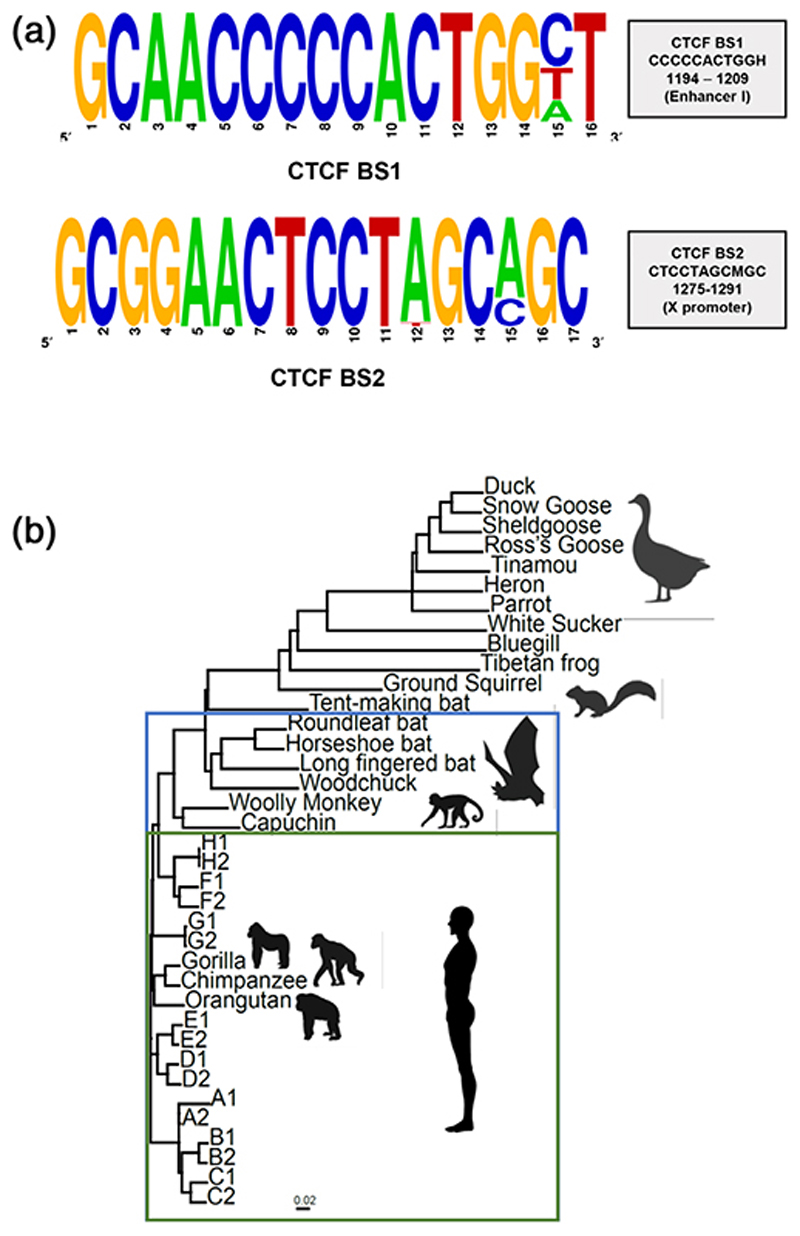
Identification of conserved CTCF binding sites in HBV genomes and diverse Hepadnaviridae (a) Conservation of CTCF BS among 7,313 HBV sequences (HBVdb.fr). All sites, except where indicated, are >98% conserved. (b) Neighbour-joining phylogenetic tree of members of the *Hepadnaviridae* (adapted from McNaughton et al., 2019). The green box shows viral genomes that encode both CTCF BS1 and 2 (all human and old world primate viruses), whereas the blue box shows viral genomes encoding only CTCF BS1 (new world monkeys, woodchucks and all bats except the tent making bat)

To investigate a role for CTCF in the HBV life cycle, we first assessed whether the viral genome encodes any CTCF binding sites, using an open access database (http://insulatordb.uthsc.edu/), to identify putative binding sites. Screening >7,000 HBV sequences available in the HBV database (Hayer et al., 2013) identified two CTCF binding sites (BS) between nucleotides 1194-1209 in EnhI (CTCF BS1) and 1275-1291 in the Xp (CTCF BS2). Importantly, these binding sites are conserved among all HBV genotypes (Figure 1a). The *Hepadnaviridae* family includes a number of related viruses that infect other species, including birds, mammals, fish, reptiles and amphibians. Inspection of reference sequences from distinct *Hepadnaviridae* showed that both consensus CTCF binding sites are conserved in viruses infecting primates and the majority of mammals and bats but are absent from viruses infecting birds, fish or amphibians, demonstrating evolutionary conservation of both CTCF binding sites (Figure 1b).

**Figure 2:**
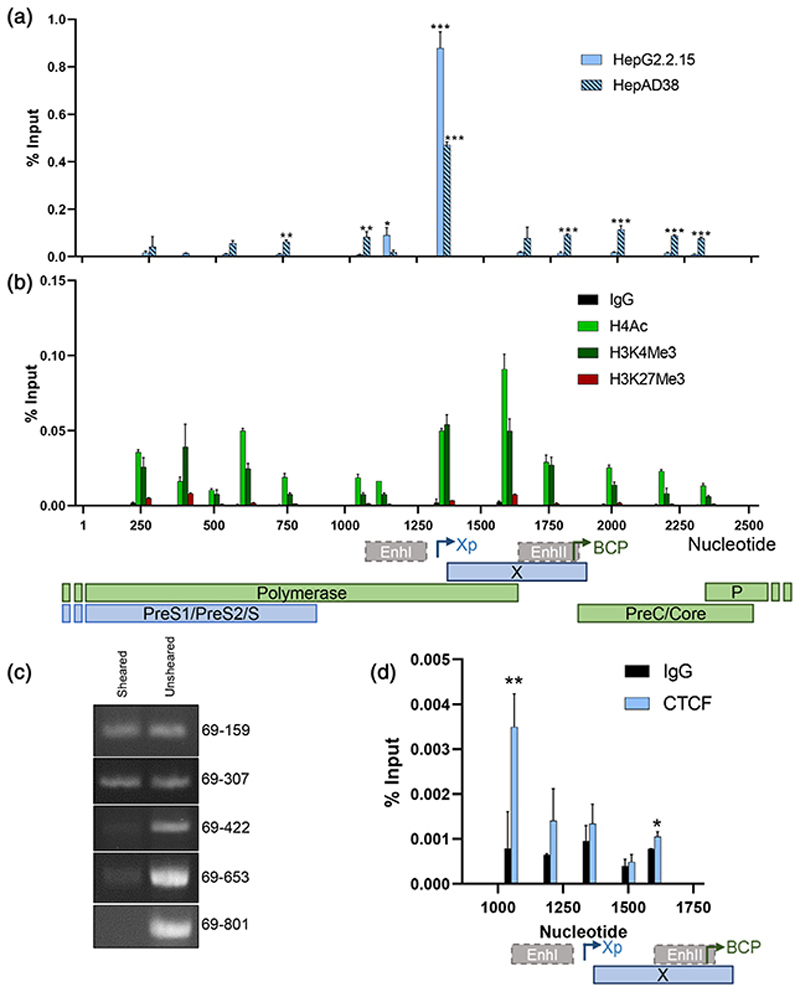
CTCF associates with HBV DNA and is enriched at viral Enhancer I and X promoter. (a) Association of CTCF with HBV DNA in HepG2.2.15 and HepAD38 cells was analysed by ChIP-qPCR and presented as % Input recovery. Statistical significance shows comparison of CTCF-specific ChIP with maximal recovery using IgG control (dotted line). (b) The distribution of histone modifications (H3K4Me3, H3K27Me3 and H4Ac) in HepG2.2.15 cells by ChIP-qPCR. (c) Chromatin shearing in HepG2-HBV-Epi cells was assessed by PCR of sonicated versus nonsonicated chromatin. Amplicons were generated with a constant sense primer (anneals at nt 69) and anti-sense primers binding at increasing distance from the sense primer (nt 159, 307, 422, 653 and 801). Amplification of HBV DNA was assessed by SyBr green staining of bands separated by electrophoresis. (d) Association of CTCF was assessed by ChIP-qPCR. (A, B and D) Data shown are the mean ± *SEM* of three technical repeats and are representative of three biological repetitions. *p* values were determined using a paired *t* test. *denotes *p* < .05, **denotes *p* < .01, ***denotes *p* < .001. Annotation of HBV genome features, including open reading frames, enhancers and selected promoters, is shown below the histograms

To assess whether these putative motifs can bind CTCF, we selected two independent lines, HepG2.2.15 (Sells et al., 1987) and HepAD38 (Ladner et al., 1997), that carry integrated copies of HBV genomes, maintain cccDNA and generate infectious virus. We isolated chromatin from nuclear fractions to limit contamination of cytoplasmic rcDNA and performed anti-CTCF chromatin immunoprecipitation (ChIP) followed by quantitative PCR (ChIP-qPCR). Primers were selected to amplify 100-200 base pair regions of the viral genome to provisionally identify CTCF binding sites. We show low-level CTCF binding above the control IgG across the viral DNA with a significant enrichment in the Xp in both cell lines (Figure 2a), consistent with our motif scanning results. These data are in line with earlier reports for CTCF binding cellular target genes (Zhang et al., 2017). Analysing histone modifications of HBV chromatin purified from HepG2.2.15 cells showed minimal evidence for the repressive H3K27Me3, which is in agreement with previous reports (Flecken et al., 2019; Tropberger et al., 2015) (Figure 2b). In contrast, ChIP for histone marks associating with active transcription, including H4Ac and H3K4Me3, identified these epigenetic marks throughout the viral genome, with an enrichment in the BCP and Xp regions (Figure 2b).

Since both HepG2.2.15 and HepAD38 cell lines carry integrated viral genomes and cccDNA, we are unable to discriminate CTCF binding between these forms of viral DNA. We studied HepG2 cells expressing an episomal copy of HBV DNA (HepG2-HBV-Epi) (Lucifora et al., 2014) to evaluate whether CTCF can bind episomal nonintegrated copies of HBV DNA. Our initial experiments assessed whether our sonication protocol sheared HBV DNA by PCR amplification using primers for viral targets of increasing length pre- and postsonication. While the unsheared chromatin yielded a series of PCR products of increasing length, only amplicons below 238 base pairs were detected in the sonicated material (Figure 2c). Amplicons over 353 base pairs were barely visible in the sonicated samples, demonstrating effective shearing of episomal HBV genomes. ChIP of sheared chromatin, isolated from HepG2-HBV-Epi nuclear extracts, showed CTCF bound to the EnhI region (Figure 2d). We noted relatively lower ChIP of viral DNA from the HepG2-HBV-Epi cells compared to HepG2.2.15 or HepAD38 cells, which may reflect differences in the epigenetic status of the viral DNA in these model systems. Our observation that CTCF binds EnhI, the major transcriptional regulatory element of the BCP and Xp, suggests that CTCF regulates its activity.

### 2.2 CTCF represses HBV enhancer

**Figure 3:**
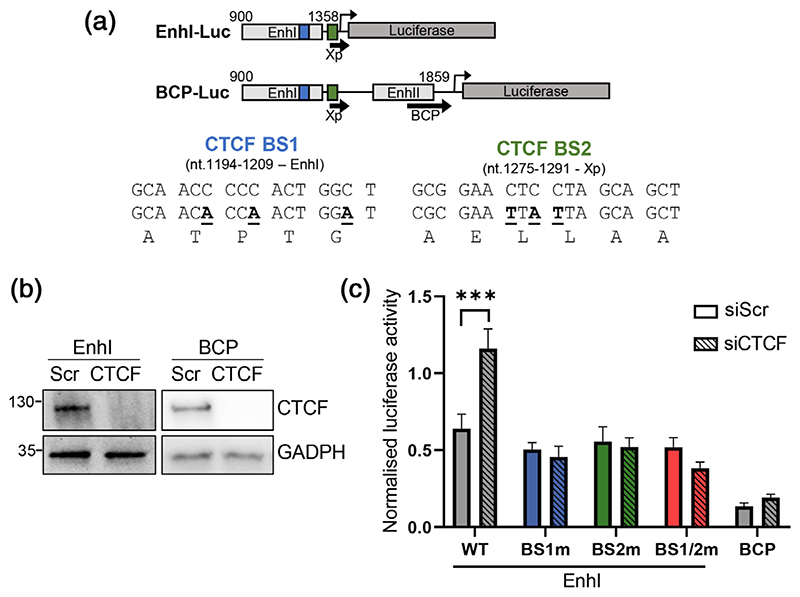
CTCF represses HBV Enhancer I activity. (a) Depiction of HBV genome regions cloned upstream of Firefly luciferase in transcriptional reporter plasmids and mutagenesis strategy of CTCF BS1 and BS2 showing viral enhancers, Xp and BCP, and CTCF BS 1 (blue) and CTCF BS 2 (green). (b) Western blot showing depletion of CTCF after siRNA transfection in pEnhI-Luc and pBCP-Luc transfected HepG2-NTCP cells. (c) Firefly luciferase activity normalised to *Renilla* luciferase expression in HepG2-NTCP cells co-transfected with pGL3-basic, pEnhI-Luc or pBCP-Luc and either scrambled (Scr) or CTCF-specific siRNA duplexes. Normalised luciferase activity in HepG2-NTCP cells transfected pEnhI-Luc containing mutations in CTCF binding site 1 (BS1m) or 2 (BS2m) or a combination of both (BS1/2m). Data shown are the mean ± *SEM* of three independent repetitions. *p* values were determined by the Sidak’s ANOVA multiple comparisons test. ***denotes *p* < .001

To analyse the role of CTCF in regulating HBV enhancer activity, we used promoter constructs encoding Firefly luciferase under the control of EnhI and Xp (nt 900-1358) or the BCP (nt 900-1859) (Figure 3a) (Ko, Lee, Windisch, & Ryu, 2014). We silenced CTCF in HepG2-NTCP using an siRNA Smartpool (Figure 3b) and transfected the viral promoter plasmids along with a *Renilla* luciferase control plasmid and measured the activity after 72 hr. Transient knock-down of CTCF protein significantly increased HBV EnhI activity (Figure 3c). To assess whether the putative CTCF directly regulated EnhI, we introduced silent mutations into the pEnhI-Luc to abrogate CTCF binding (Schmidt et al., 2012), without altering the polymerase protein sequence as this would adversely affect subsequent experiments with intact HBV genomes (Figure 3a). Mutation of either CTCF BS1 (BS1m) or BS2 (BS2m) in isolation or in combination (BS1/2m) abrogated the increase in EnhI activity after CTCF depletion (Figure 3c). Silencing CTCF protein showed a minimal effect on the BCP activity, suggesting that CTCF represses EnhI but this effect may be blunted in the presence of an NRE and overall reduced activity in the full transcriptional reporter construct (Figure 3c). Together, these data suggest that CTCF binds to both motifs within EnhI to directly repress its activity.

### 2.3 Silencing CTCF increases HBV preC/pgRNA levels

**Figure 4:**
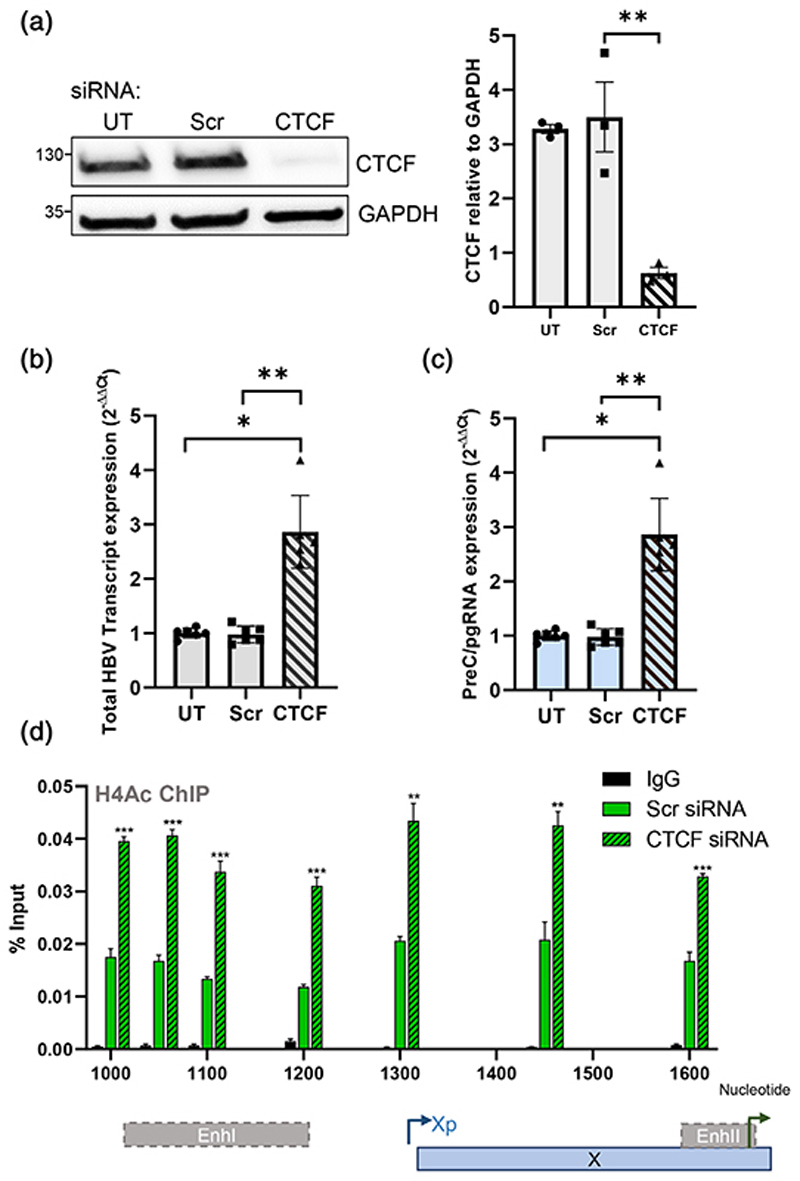
CTCF represses preC/ pgRNA transcription from HBV cccDNA. (a) HepG2-HBV-Epi cells were untransfected (UT) or transfected with scrambled (Scr) or CTCF-specific siRNA duplexes and incubated for 72 hr. CTCF depletion was assessed by western blotting and quantification in three independent experiments shown. (b) Total viral RNA and (c) preC/pgRNA levels were quantified by qPCR as previously described (D’Arienzo et al., 2019). Data are the mean ± SD of two independent experiments performed in triplicate. *p* values were determined by the Kruskal-Wallis ANOVA multiple group comparison. (d) Enrichment of H4Ac marks was assessed by ChIP-qPCR and shown as % Input recovery. *p* values were determined using a paired t test. *denotes *p* < .05, **denotes *p* < .01, ***denotes *p* < .001

To determine the effect of CTCF depletion on viral transcripts, we selected to use the HepG2-HBV-Epi cells as we previously demonstrated CTCF binding to the viral genome in these cells. We confirmed effective knock-down of CTCF at the protein and RNA level 72 hr post-siRNA transfection (Figure 4a). We observed a significant increase in total HBV transcripts and preC/pgRNA levels after CTCF depletion (Figure 4b,c). To determine whether the observed increase in preC/pgRNA levels was due to an alteration of the HBV epigenome after CTCF depletion, we measured H4Ac modification of viral DNA as this was previously reported to associate with HBV transcription (Pollicino et al., 2006). Silencing of CTCF in HepG2-HBV-Epi cells increased H4Ac abundance within the viral enhancers, BCP Xp and BCP, suggesting that CTCF regulates the epigenetic status of HBV cccDNA (Figure 4d).

**Figure 5:**
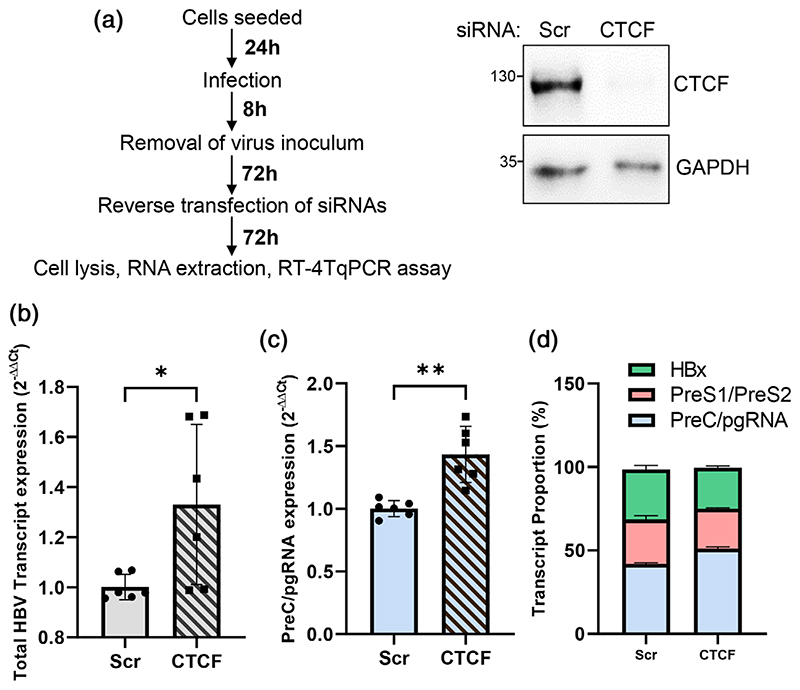
CTCF represses HBV preC/pgRNA transcription in de novo infected HepG2-NTCP cells. (a) HBV infected HepG2-NTCP were transfected with scrambled (Scr) or CTCF-specific siRNA duplexes and cultured for 72 hr and CTCF depletion assessed by western blotting. (b) Total HBV transcript abundance, (c) preC/pgRNA levels and (d) the relative proportion of individual HBV transcripts were analysed by PCR as previously described (D’Arienzo et al., 2019). Data are the mean ± SD of two independent experiments performed in triplicate. p values were determined using the Mann-Whitney *U* test (two group comparisons). *denotes *p* < .05, **denotes *p* < .01

To extend our studies and to validate a role for CTCF in repressing viral transcription during a de novo infection, we silenced CTCF in HBV infected HepG2-NTCP cells (Figure 5a). Efficient depletion of CTCF was demonstrated by western blotting (Figure 5a), and viral RNAs were analysed by RT-qPCR. In agreement with our earlier data with HepG2-HBV-Epi cells, CTCF depletion in this de novo infection model increased total transcripts and preC/pgRNA levels (Figure 5b,c). We previously reported a qPCR technique to quantify the relative abundance of HBV RNAs (D’Arienzo et al., 2019), and we used this method to assess the effect of CTCF silencing on the pattern of viral RNAs, showing no significant differences in the pattern of preSl, preS2 and HBx RNAs (Figure 5d). These data support a model where CTCF represses HBV cccDNA transcription, the major transcriptional template in de novo infected HepG2-NTCP cells. Taken together, our findings provide evidence that CTCF represses the BCP activity and hence preC/pgRNA levels.

### 2.4 Mutation of CTCF binding sites within HBV enhancer I increases transcription

**Figure 6:**
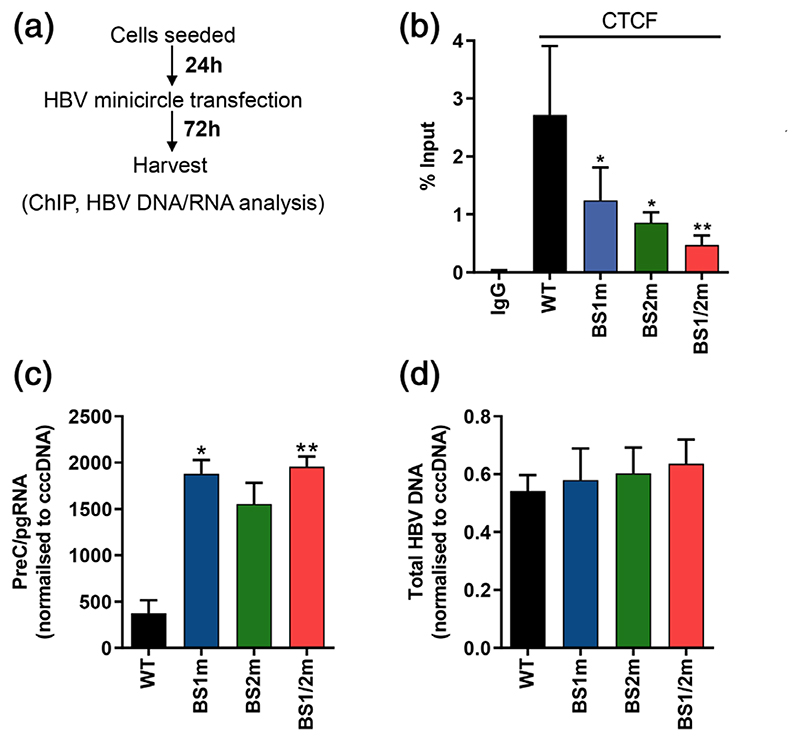
Mutation of CTCF binding sites in HBV mcDNA results in increased preC/pgRNA levels. (a) HepG2-NTCP cells were transfected with wild type HBV mcDNA (WT) or mcDNA with CTCF binding 1 (BS1m) or 2 (BS2m) or both sites mutated in combination (BS1/2m). (b) Cells were harvested 72 hr post transfection and CTCF binding analysed by ChIP-qPCR and presented as % of enrichment relative to input chromatin (% input). (c) preC/pgRNA and levels were quantified by qRT-PCR and normalised to cccDNA amount per cell and (d) total HBV DNA levels were quantified by qRT-PCR and normalised to cccDNA amount per cell to determine mcHBV transfection efficiency. Data are the mean ± *SEM* of at least three independent experiments. *p* values were determined using the Kruskal-Wallis ANOVA multiple group comparison. *denotes *p* < .05, **denotes *p* < .01

To demonstrate a direct role for CTCF binding to, and regulating, cccDNA transcription, we utilised the HBV minicircle (mcHBV) technology, as a model to study cccDNA transcription and replication (Yan et al., 2017). We mutated CTCF BS1 and BS2 alone or in combination in the mcHBV as described in Figure 3a. HepG2-NTCP cells were transfected with wild-type mcHBV (WT) or mutant mcHBV; BS1m, BS2m or BS1/2m, and harvested 3 days after transfection (Figure 6a). Analysis of CTCF binding by ChIP revealed that mutation of BS1 or BS2 alone significantly reduced CTCF binding by over >75% with the combined mutation resulting in an almost complete loss of CTCF-mcHBV complexes (Figure 6b). qPCR analysis showed a significant increase in preC/pgRNA levels when either or both of the CTCF BS were mutated (Figure 6c). However, no differences were observed in HBV mcDNA levels, confirming comparable transfection efficiencies (Figure 6d). These data provide evidence of direct recruitment of CTCF to HBV DNA and show a repressive role for CTCF in regulating HBV transcription.

## 3 Discussion

In this study, we identified two CTCF-binding motifs within transcription regulatory elements, EnhI and Xp, of the HBV genome. We demonstrate CTCF binding to HBV DNA by ChIP-qPCR in the region of these binding sites using various model systems that bear both integrated genomes and a cccDNA pool, or cells exclusively expressing episomal copies of viral DNA. Our sonication method sheared cccDNA-like episomes and demonstrated CTCF binding to EnhI, albeit slightly upstream of the peak enrichment of CTCF binding in the integrant lines. This altered location of CTCF binding could reflect differential usage of CTCF binding sites in the different model systems or could reflect less efficient shearing of cccDNA-like molecules compared to integrated HBV DNA. Nonetheless, our ChIP-qPCR experiments allowed provisional mapping of CTCF binding sites that were confirmed by mutagenesis studies using promoter reporter constructs and mcHBV DNA. Importantly, these CTCF binding sites are conserved among all HBV genotypes and across the wider *Hepadnaviridae* family, consistent with an evolutionary conserved role in the replication of these viruses. Finally, we show a role for CTCF to repress HBV transcription.

Using several complementary HBV replication models, we show that siRNA depletion of CTCF and mutation of CTCF binding sites significantly increased preC/pgRNA levels, consistent with a role for CTCF in repressing viral transcription. To understand the mechanism of CTCF action, we used transcriptional reporter assays and found that silencing CTCF significantly increased EnhI activity. Furthermore, mutating the CTCF BS within EnhI attenuated this phenotype, confirming a direct role for CTCF in regulating EnhI. However, analysis of the full BCP, containing both EnhI and EnhII, revealed that the phenotype of CTCF silencing was lost. It is likely that the attenuation of BCP activity after CTCF silencing is explained by the dominant repressive effects of the NRE within EnhII, highlighting the context-dependent activity of CTCF in regulating HBV. However, increased activity of the BCP is observed after CTCF silencing in cells containing the full viral episome, which may reflect differential chromatinization and epigenetic modification of the transcriptional reporters as compared to the full viral episome. Alternatively, the transcriptional elements in isolation are no longer subject to regulation by distal elements contained within the intact episome. While the transcriptional reporters used in this study provide a useful tool in the initial characterisation of CTCF function in HBV EnhI modulation, the results obtained in the context of a chromatinised viral episome may better reflect the role of CTCF in the HBV infection cycle.

To confirm a direct role of CTCF in repressing HBV transcription, we transfected HepG2-NTCP cells with mcHBV mutated in the CTCF BSs. Although the extent to which we could mutate CTCF BS was limited, to maintain the amino acid sequence of the polymerase, we observed a significant reduction of CTCF binding to mcHBV, lacking either BS1 or BS2, or both sites mutated in combination. These studies identify CTCF BSs within the viral genome and confirm CTCF association with HBV DNA. Consistent with the increased preC/pgRNA levels observed in two HBV replication model systems after CTCF depletion, we observed a significant increase in preC/ pgRNA when CTCF BS1 was mutated. A similar increase in preC/ pgRNA was observed when CTCF BS2 was mutated, although this did not reach statistical significance. While the mutation of both BS showed a significant increase in preC/pgRNA abundance, suggesting these sites do not function in a synergistic manner within this model system.

Aberrant reverse transcription of pgRNA can generate doublestranded linear DNA that can integrate into the host genome (Tu, Budzinska, Shackel, & Urban, 2017). This integration step is not part of the productive HBV life cycle and occurs at a low frequency (<1 copy per diploid host genome in infected tissues) (Podlaha et al., 2019). Since integrated copies of HBV DNA generally lack a functional basal core promoter and associated CTCF binding sites, we would anticipate a minimal role for CTCF in directly regulating integrant derived transcripts. HBV integration can cause host genomic instability leading to tumour progression through tumour suppressor gene inactivation and/or oncogene activation (Zhao et al., 2016). Oncogenic integration events are thought to provide a growth advantage to cells, inducing tumourigenesis. HBV integration occurs at random sites, although a preference for integration within regions of open chromatin has been reported (Furuta et al., 2018). It will be interesting to determine whether integration of HBV DNA into the host results in an alteration of local chromatin interactions and host cell gene regulation by the insertion of a virally encoded CTCF binding site(s), as reported for the human retrovirus, HTLV-1 (Melamed et al., 2018). Such genomic rearrangements could have an impact on host cell gene expression and contribute to HBV-driven carcinogenesis.

Analysing the epigenetic status of HBV DNA in HepG2.2.15 hepatoma cells revealed a lack of the repressive H3K27Me3 and enrichment of epigenetic marks associated with active transcription in the BCP and Xp regions, downstream of the CTCF binding sites. We noted a similar enrichment of H4Ac in episomal DNA in HepG2-HBV-Epi cells. These findings are consistent with previous reports studying the epigenetic status of HBV cccDNA in various model systems and liver biopsy samples (Flecken et al., 2019; Tropberger et al., 2015). Silencing of CTCF resulted in an increase in H4Ac abundance in HBV cccDNA, which associates with increased HBV preC/pgRNA levels. CTCF has been reported to directly repress transcription via recruitment of the Sin3/histone deacetylase compressor complex resulting in reduced histone acetylation (Lutz et al., 2000) that may explain these observations.

Taken together, these findings suggest that CTCF represses HBV transcription by insulating the BCP from the upstream enhancer element. EnhI is an important regulator of all HBV promoters and is essential for viral transcription (Hu & Siddiqui, 1991). In support of this, HBV-transgenic mice, lacking EnhI, are defective in virion production (Guidotti, Matzke, Schaller, & Chisari, 1995). The repression of EnhI by CTCF is likely to have a significant impact on the virus life cycle and reduce particle genesis and may therefore limit cccDNA pools. To assess whether infection perturbs CTCF expression, we analysed a publically available Affymetrix microarray database from chronic HBV infected patients (Zhou et al., 2017). We observed comparable CTCF transcript levels in normal and chronic HBV infected samples (Figure S1) with no evidence for HBV infection to perturb intra-hepatic CTCF transcript levels.

Analysis of the genomic distribution of CTCF BS in the human genome suggests a similar enhancer-blocking activity of CTCF as numerous CTCF binding loci are situated between known transcriptional enhancers and associated promoter elements (Xie et al., 2007). Such enhancer-blocking activity has been extensively characterised at imprinted loci, such as the insulin-like growth factor 2 (IGF2)/H19 locus and in development at the β-globin locus (Bell, West, & Felsenfeld, 1999; Chung, Whiteley, & Felsenfeld, 1993). CTCF regulates herpes simplex virus differential transcriptional programmes during the lytic and latent phases of the viral life cycle through its enhancer-blocking activity (Washington et al., 2018). Our previous work with HPV showed that CTCF repressed transcription by stabilising an epigenetically repressed chromatin loop between the viral proximal enhancer and a distal CTCF binding site. However, this repression was not associated with direct binding of CTCF to the HPV enhancer, suggesting that HBV and HPV have evolved fundamentally different mechanisms of CTCF-dependent transcriptional repression.

## 4 Experimental Procedures

### 4.1 Cell lines and antibodies

HepG2.2.15 (Sells, Chen, & Acs, 1987), HepAD38 (Ladner et al., 1997), HepG2-HBV-Epi (Lucifora et al., 2014) and HepG2-NTCP cells were maintained in Dulbecco’s Modified Eagles Medium (DMEM, #31966) supplemented with 10% fetal bovine serum (FBS), 2 mM L-glutamine, 1 mM sodium pyruvate, 50 U/ml penicillin/streptomycin and non-essential amino acids (all reagents from Invitrogen, UK). All cells were maintained in a 5% CO_2_ atmosphere at 37°C. HepG2-HBV-Epi cells were kept at low passage to limit HBV DNA integration. The following primary antibodies were used: anti-CTCF (#61311), anti-H3K4Me3 (#39915), anti-H3K27Me3 (#39155) and anti-H4Ac (#39925) were all purchased from Active Motif (UK) and anti-GAPDH (SC-32233) was purchased from Santa Cruz.

### 4.2 ChIP and quantitative PCR

HepG2.215, HepAD38 cells or HepG2-HBV-Epi cells were fixed with 1% formaldehyde (Sigma Aldrich) for 10 min at room temperature before quenching with 125 mM glycine. The cells were washed with ice cold PBS, containing EDTA-free protease inhibitors (Roche) and 5 mM sodium butyrate, and frozen at −80°C. Pellets were resuspended in ChIP lysis buffer (Active Motif) supplemented with protease inhibitors and incubated on ice for 30 min. Cells were dounced 30 times using the tight pestle to release nuclei and centrifuged at 2500g for 10 min at 4°C. The supernatant was removed and discarded. Nuclei were re-suspended in shearing buffer (Active Motif) pulse sonicated using a Sonics Vibra Cell CV18 sonicator fitted with a micro-probe at 25% amplitude for 15 min on ice using 30 s on/off cycles. Chromatin samples were cleared by centrifugation and stored at −80° C.

Sonication of HBV cccDNA was evaluated by conventional PCR amplification of increasing amplicon size using a constant sense primer and anti-sense primers described in Table 1. Phenolchloroform extracted DNA from HepG2-HBV-Epi cells before and after sonication was quantified using a NanoDrop ND-1000 spectrophotometer. PCR reactions included 100 ng DNA, MyTaq Red PCR Mix (Bioline) and 200 nM sense/anti-sense primers and amplification following 35 cycles of 95°C, 15 s; 55°C, 15 s; 72°C, 30 s assessed by agarose gel electrophoresis. Products were visualised using SyBr Green Safe dye (Invitrogen).

For ChIP, sonicated lysates were clarified by centrifugation at 16,000*g* for 10 min and CTCF or histone complexes immunoprecipitated with 5-8 μg antibody using a ChIP-IT® Express Chromatin Immunoprecipitation kit, including Protein A magnetic beads as per manufacturer’s instructions (Active Motif, USA). The input and immunoprecipitated DNA were quantified by real-time PCR using a Stratagene MX3500P PCR System. The values were calculated as % recovery respective to input DNA signals. All oligonucleotide sequences are listed in Table 1.

### 4.3 siRNA transfection

Cells were trypsinized to reverse transfect with 25 nM of CTCF-specific or scrambled TARGETplus Smartpool siRNAs (Horizon, USA) using DharmaFECT4 (20% of amount recommended by the manufacturer’s protocol; ThermoFisher). Cells with no siRNA (un-treated; UT) were also assayed to assess lethality of CTCF depletion.

### 4.4 SDS-PAGE and western blots

Cells were lysed in urea lysis buffer (8 M urea, 150 mM NaCl, 20 mM Tris, pH 7.5, 0.5 M β-mercaptoethanol) supplemented with protease inhibitor cocktail (Roche) and sonicated for 10 s at 20% amplitude using a Sonics Vibra Cell sonicator fitted with a micro-probe. Following quantification of protein concentration by Bradford assay, samples were diluted in Lameli buffer before incubating at 95°C for 5 min. Proteins were separated on a 10% polyacrylamide gel and transferred to PVDF membranes (Amersham). The membranes were blocked in TBS-T, 5% skimmed milk, and proteins were detected using specific primary (diluted at 1:1000) and HRP-secondary antibodies (ThermoFisher, diluted at 1:10,000). Protein bands were detected using Pierce SuperSignal West Pico chemiluminescent substrate kit (Pierce) and images were collected using a Fusion FX Imaging system (Peqlab).

### 4.5 HBV transcription reporter assays

1 × 10^5^ HepG2-NTCP cells were seeded in collagen-coated 24-well plates. Immediately following cell seeding, transfection mixes were added, containing 100 ng of either pGL3b-EnhI, pGL3b-BCP or pGL3b-basic, 25 ng *Renilla* luciferase control plasmid (pCMV-Renilla), 25 nM scrambled or CTCF-specific siRNA and 1.5 μl Lipofectamine RNAiMAX™ (ThermoFisher) in 100 μl OptiMEM (ThermoFisher). Cells were incubated at 37°C, 5% CO_2_ for 72 hr before being washed with PBS and 200 μl Passive Lysis buffer (Promega) added to each well. Samples were incubated at RT for 30 min with gentle rocking. Lysates were cleared by centrifugation and 20 μl of each added to a white 96-well microtiter plate. FireFly and *Renilla* luciferase activity were detected using the Dual-Luciferase® Reporter Assay (Promega) using a GloMAX®-Multi Detection system (Promega). 50 μl reagent added at a speed of 200 μl/s followed by mixing and 2 s delay. Integration time was 10 s with 1 read/well for Firefly luciferase detection. The same protocol was used for subsequent *Renilla* luciferase detection. Normalised luciferase activity was calculated by dividing Firefly luciferase activity by *Renilla* luciferase activity.

### 4.6 HBV de novo infection

Purified HBV was produced from HepAD38 cells as previously reported (Ko et al., 2018). HepG2-NTCP cells were seeded on collagen-coated plasticware and infected with HBV at an MOI of 250 genome equivalents per cell in the presence of 4% PEG 8,000. Viral inoculum was removed 8 hr after infection, by extensive washing with PBS, and cells were maintained in DMSO-free DMEM/10% FBS.

### 4.7 RNA isolation for cDNA synthesis

Total cellular RNA was extracted using an RNeasy mini kit (Qiagen) following the manufacturer’s protocol. To remove any residual HBV DNA, the samples were treated with RNAse-free DNase I (14 Kunitz units/ rxn, Qiagen) for 30 min at RT. RNA concentration and quality were assessed using a NanoDrop 1,000 spectrophotometer (ThermoFisher) and 2,100 Bioanalyzer (Agilent). cDNA synthesis was performed with 0.25-1 μg of RNA in a 20 μl total reaction volume using a random hexamer/oligo dT strand synthesis kit as per the manufacturer’s instructions (10 min at 25°C; 15 min at 42°C; 15 min at 48°C; SensiFast, Bioline). All oligonucleotide sequences are listed in Table 1.

### 4.8 Quantitative PCR of HBV transcripts

All PCR reactions were performed using a SYBR green real-time PCR protocol (qPCRBIO SyGreen, PCR Biosystems) in a Lightcycler 96™ instrument (Roche). The amplification conditions were: 95°C for 2 min (enzyme activation), followed by 45 cycles of amplification (95°C for 5 s; 60°C for 30 s). A melting curve analysis was performed on the completed reactions to assess specificity and purity of the amplicons (95° C for 10 s; 60°C for 60 s; followed by gradual heating from 60 to 97°C at 1°C/s). DNase-treated RNA samples that had not been reverse-transcribed were amplified to verify the absence of residual DNA contamination. All oligonucleotide sequences are listed in Table 1.

### 4.9 HBV mcDNA purification and transfection into cells

The plasmid, pMC-HBV, contains the 1.0 HBV genome (awy) and has been previously described (Yan et al., 2017). CTCF BS1 and CTCF BS2 were mutated by site-directed PCR mutagenesis using the primers detailed in Table 1 and Prime Star Max (Takara) mutagenesis kit following the manufacturer’s protocols and confirmed by sequencing. ZYCY10P3S2T competent bacteria (System Bioscience) were then transformed with the pMC-HBV (WT, BS1m, BS2m or BS1/2m) and a single colony amplified in Terrific Broth overnight at 37°C. Two volumes of LB medium supplemented with 0.04 N NaOH and 0.02% L-Arabinose were added to the culture and further incubated for 8 hr at 37°C. Plasmid DNA was extracted using the Nucleobond Xtra Maxi kit according to the manufacturer’s protocol (Macherey-Nagel) and digested with *Ndel* (New England Biolabs) for 2 hr at 37°C and plasmid-safe DNase (System Bioscience) overnight at 37°C. After purification, plasmid DNA was assessed by agarose gel electrophoresis to check for elimination of the parental plasmid. HepG2-NTCP cells at 80-90% confluency were transfected with the pMC-HBV plasmids using TransIT-2020 (Mirus) according to the manufacturer’s protocol in DMEM supplemented with 5% FBS, 1% Glutamax and 1% sodium pyruvate. The following day, cells were washed once with PBS and cultured for 72 hr in DMEM supplemented with 5% FBS, 1% Glutamax, 1% sodium pyruvate and 1% penicillin/streptomycin.

### 4.10 HBV nucleic acid quantification from mcHBV-transfected cells

Total DNA was extracted using MasterPure™ Complete DNA Purification Kit (Epicentre). Total RNA was extracted using ExtractAll TRI-Reagent (Sigma Aldrich), precipitated in isopropanol, washed in ethanol and re-suspended in RNase-free water. Extracted RNA was digested with RNAse-free DNase I (Qiagen) and cDNA synthesised using SuperScript Ill reverse transcriptase (Invitrogen). cccDNA was quantified after *ExoI* + *ExoIII* endonuclease (Epicentre) digestion of total extracted DNA for 2 hr at 37°C, followed by 20 min inactivation at 80°C. Real-time qPCR for total HBV DNA and cccDNA was performed using an Applied QuantStudio 7 machine (BioSystem) and TaqMan Advanced Fast Master Mix. Total HBV DNA was quantified using the TaqMan assay Pa03453406_s1; cccDNA specific primers and probes were: forward 5’-CCGTGTGCACTTCGCTTCA-3’; reverse 5’-GCACAGCTTGGAGGCTTGA-3’ TaqMan probe [6FAM] CATGGAGACCACCGTGAACGCCC[BBQ] (Testoni et al., 2019). Serial dilutions of a plasmid containing an HBV monomer (pHBV-EcoRI) served as quantification standard for total HBV DNA and cccDNA. The number of cellular genomes was determined by using the β-globin TaqMan assay Hs00758889_s1 (ThermoFisher). preC/pgRNA was quantified using the following primers and probe: forward 5’-GGAGTGTGGATTCGCACTCCT-3’; reverse 5’-AGATTGAGATCT TCTGCGAC-3^’^ and TaqMan probe [6FAM]AGGCAGGTCCCCTAGAA GAAGAACTCC[BBQ] (Testoni et al., 2019). Relative amount was normalised over the expression of housekeeping gene GUSB (Hs99999908_m1).

### 4.11 Chromatin immunoprecipitation from infected HepG2-NTCP cells and mcHBV-transfected cells

Seventy two hours after de novo infection or mcHBV transfection, cells were washed twice with PBS and cross-linked with 1% formaldehyde for 10 minutes at 37°C. After 5 min of quenching with 125 mM glycine at 37°C, cells were washed twice with PBS, centrifuged for 5 min at 300*g* and incubated with Nuclear Lysis Buffer (5 mM PIPES, 85 mM KCl, 0.5% NP-40) for 30 min on ice to isolate nuclei. The lysate was then dounced 10 times and centrifuged for 5 min at 800*g* at 4°C. Nuclear membranes were then broken by two cycles of sonication 30 s ON, 30 s OFF on a Bioruptor (Diagenode). Debris were pelleted 10 min at 11,000*g* at 4°C. The supernatant was diluted 10 times with RIPA buffer (10 mM Tris-HCl pH 7.5, 140 mM NaCl, 1 mM EDTA, 0.5 mM EGTA, 1% Triton X-100, 0.1% SDS, 0.1% Na- deoxycholate) supplemented with Complete Mini EDTA-free protease inhibitor (Roche Diagnostics) and 1 mM PMSF and pre-cleared for 2 hr at 4°C by adding magnetic Protein G Dynabeads (Life Technologies). Beads were discarded and 1 μg of anti-CTCF antibody (Diagenode #C15410210) or isotype-matched negative control were added to the chromatin. After an overnight incubation at 4°C, magnetic Protein G Dynabeads and samples were incubated for 2 hr at 4°C with agitation. Beads were washed five times with RIPA buffer, once with TE buffer and re-suspended in Elution buffer (20 mM Tris-HCl pH 7.5, 5 mM EDTA, 50 mM NaCl, 1% SDS, 50 μg/ml proteinase K). Chromatin was reverse cross-linked by incubation at 68°C for 2 hr and purified by phenol:chloroform:isoamyl alcohol 25:24:1 (Life Technologies) extraction and ethanol precipitation. cccDNA was quantified using the primers and probes listed above (Testoni et al., 2019).

## Supplementary Material

Supporting information

## Figures and Tables

**Table 1:** Detailing all primer sequences used

Primer pair	Forward (5’-3’)	Reverse (5’-3’)
T1	GGGGAACTAATGACTCTAGCTACC	TTTAGGCCCATATTAGTGTTGACA
T2	CAAGGTAGGAGCTGGAGCATTC	GAGGCAGGAGGCGGATTTG
T3	CTCCAGTTCAGGAACAGTAAACCC	AGGAATCCTGATGTGATGTTCTCC
T4	ACGGGGCGCACCTCTCTTTA	GTGAAGCGAAGTGCACACGG
β-actin	CCAACCGCGAGAAGATGA	CCAGAGGCGTACAGGGATAG
**Mutagenesis primers (Luc)**		
CTCF BS1	CTGGATGGGGCTTGGTCATGCGC	TTGGTGTTGCGTCAGCAAACACTTGG
CTCF BS2	AGCAGCTTGTTTTGCTCGCAGC	AATAATTCCGCAGTATGGATCGG
**Mutagenesis primers (pMC-HBV)**		
CTCF BS1	GTGTTTGCTGACGCAACACCAACT GGATGGGGCTTGGTC	GACCAAGCCCCATCCAGTTGGTGTTGCGT CAGCAAACAC
CTCF BS2	GCCGATCCATACTGCGGAATTATT AGCAGCTTGTTTTGCTCGCAGCAGG	CCTGCTGCGAGCAAAACAAGCTGCTAATA ATTCCGCAGTATGGATCGGC
**HBV ChiP primers**		
178-307	TTCCTAGGACCCCTTCTCGT	GGCCAAGACACACGGTAGTT
254-428	TCGTGGTGGACTTCTCTCAA	TGAGGCATAGCAGCAGGAT
346-422	TCCTGTCCTCCAACTTGTCC	AGCAGCAGGATGAAGAGGAA
462-562	GTTGCCCGTTTGTCCTCTAATTC	GGAGGGATACATAGAGGTTCCTTGA
518-653	GCCGAACCTGCATGACTACT	GCCGAACCTGCATGACTACT
718-801	CCCACTGTTTGGCTTTCAGT	CAGCGGTAAAAAGGGACTCA
995-1108	ACGAATTGTGGGTCTTTTGG	GTTGGCGAGAAAGTGAAAGC
1089-1154	GCTTTCACTTTCTCGCCAAC	AACGGGGTAAAGGTTCAGGT
1305-1438	AGCAGGTCTGGAGCAAACAT	GACGGGACGTAAACAAAGGA
1581-1693	GTGCACTTCGCTTCACCTCT	GGTCGTTGACATTGCAGAGA
1738-1837	GGAGTTGGGGGAGGAGATTA	GGCAGAGGTGAAAAAGTTGC
1901-2054	GCATGGACATCGACCCTTAT	TGAGGTGAACAATGCTCAGG
2112-2297	CTGGGTGGGTGTTAATTTGG	TAAGCTGGAGGAGTGCGAAT
2279-2392	TTCGCACTCCTCCAGCTTAT	GAGGCGAGGGAGTTCTTCTT
2983-3133	ACAAGGTAGGAGCTGGAGCA	GTAGGCTGCCTTCCTGTCTG
**HBV cccDNA shearing**		
F69-R159	CTCCAGTTCAGGAACAGTAAACCC	AGGAATCCTGATGTGATGTTCTCC
R307		GGCCAAGACACACGGTAGTT
R422		AGCAGCAGGATGAAGAGGAA
R653		GCCGAACCTGCATGACTACT
R801		CAGCGGTAAAAAGGGACTCA

## Data Availability

The data that support the findings of this study are available from the corresponding author upon reasonable request.
