# Supplementary material for: The CCCTC-binding factor CTCF represses hepatitis B virus enhancer I and regulates viral transcription
Source: Cell Microbiol. Author manuscript; Available in PMC 2021 Feb 13. (PMC7116737; doi:10.1111/cmi.13274)
Supplement: Supporting information [file EMS115918-supplement-Supporting_information.pdf]

Zhang, B., Zhang, Y., Zou, X., Chan, A. W., Zhang, R., Lee, T. K.-W., … Ko, B. C. (2017). The CCCTC-binding factor (CTCF)–forkhead box protein M1 axis regulates tumour growth and metastasis in hepatocellular carcinoma. *The Journal of Pathology*, *243*(4), 418–430. https://doi.org/10.1002/path.4976

Zhao, L. H., Liu, X., Yan, H. X., Li, W. Y., Zeng, X., Yang, Y., … Wang, H. Y. (2016). Genomic and oncogenic preference of HBV integration in hepatocellular carcinoma. *Nature Communications*, *7*, 12992. https://doi.org/10.1038/ncomms12992

Zhou, W., Ma, Y., Zhang, J., Hu, J., Zhang, M., Wang, Y., … Liu, J. (2017). Predictive model for inflammation grades of chronic hepatitis B: Large-scale analysis of clinical parameters and gene expressions. *Liver International*, *37*(11), 1632–1641. https://doi.org/10.1111/liv.13427

SUPPORTING INFORMATION

Additional supporting information may be found online in the Supporting Information section at the end of this article.

How to cite this article: D'Arienzo V, Ferguson J, Giraud G, et al. The CCCTC-binding factor CTCF represses hepatitis B virus enhancer I and regulates viral transcription. *Cellular Microbiology*. 2020;e13274. https://doi.org/10.1111/cmi.13274
